# Detecting and recording cardiac murmurs in clinically healthy puppies in first opinion veterinary practice at the first health check

**DOI:** 10.1186/s13028-020-00535-1

**Published:** 2020-06-25

**Authors:** Marie Dirkje Beijken van Staveren, Viktor Szatmári

**Affiliations:** Department of Clinical Sciences, Faculty of Veterinary Medicine, Utrecht University, Yalelaan 108, 3584 CM Utrecht, The Netherlands

**Keywords:** Auscultation, Dogs, Inter-observer variability, Screening, Stethoscope

## Abstract

**Background:**

The frequency that cardiac murmurs are identified and recorded in first opinion veterinary practices at the first health check in puppies is unknown. The aims of the study were to assess the agreement between first opinion veterinary practitioners, a veterinary student and a veterinary cardiology specialist on detecting murmurs, and to establish whether abnormal auscultation findings had been recorded in the health certificates of clinically healthy puppies. The study included prospective and retrospective investigations, where the prospectively collected auscultation findings from a veterinary cardiology specialist and a trained veterinary student were compared to auscultation findings recorded by first opinion veterinary practitioners.

**Results:**

Cardiac auscultation was performed on 331 client-owned, clinically healthy dogs at two time points: at age 34–69 days by a first opinion veterinary practitioner and at age 45–76 days, on average 9 days later, by a veterinary cardiology specialist and a trained veterinary student. Agreement among the three was compared for the presence of a murmur. The degree of inter-observer agreement was evaluated using Cohen’s kappa. Auscultation findings, as noted in the pets’ passports, from 331 puppies and 43 different first opinion veterinary practices, were retrospectively reviewed and prospectively compared with auscultation findings from a veterinary cardiology specialist. Agreement between the veterinary cardiology specialist and the first opinion veterinary practitioners was poor (ϰ = 0.01) and significantly different (P < 0.001). First opinion veterinary practitioners had recorded a cardiac murmur in only 1 of the 97 puppies in which the veterinary cardiology specialist detected a murmur. Two-hundred-and-fifty-two puppies were auscultated by both the veterinary cardiology specialist and the student. Their agreement was fair (ϰ = 0.40) and significantly different (P = 0.024). The agreement between the student and a first opinion veterinary practitioner on these 252 puppies was poor (ϰ = 0.03) and significantly different (P < 0.001).

**Conclusions:**

This study shows that soft cardiac murmurs are rarely documented during the first veterinary health check in puppies by first opinion veterinary practitioners. Although soft murmurs may not be clinically relevant, finding and recording them is evidence of a carefully performed auscultation. Missing a non-pathological murmur is not of clinical importance; however, missing a pathological murmur could prove detrimental for the individual puppy.

## Background

Soft cardiac murmurs are commonly detected in clinically healthy puppies at 6–8 weeks of age, when the first veterinary health checks generally take place [1–4]. Soft murmurs can be either pathologic or non-pathologic [1]. Non-pathological cardiac murmurs are audible murmurs in the absence of structural cardiovascular anomalies, which in puppies and children tend to disappear spontaneously with age [1–6]. Pathological murmurs in puppies, however, are caused by structural cardiovascular lesions, most likely due to congenital cardiac anomalies [1, 7]. Though non-pathological murmurs have no consequences for the quality of life or life expectancy of the pet, pathological murmurs may be associated with increased morbidity and mortality.

Pet passports are official veterinary documents that contain the individual’s vaccination record and, in the country in which the present study took place, a section about the serial health status of each organ (including the heart) of the puppy. This certificate is filled out by first opinion veterinary practitioners at each veterinary health check, which typically coincides with the vaccinations. The first vaccination usually takes place at 6 weeks of age. Notes in the passport on this date are the only health-related documentation available for the new owner when the breeder is selling the puppy shortly after the first vaccination.

The aims of the present study were to assess the agreement between first opinion veterinary practitioners, a student and a veterinary cardiology specialist on detecting murmurs, and to establish whether or not abnormal auscultation findings were recorded in the health certificates of clinically healthy puppies at the initial physical examination at the age of 6–8 weeks by first opinion veterinary practitioners. An additional aim of the study was to compare the auscultation findings of a veterinary cardiology specialist and a final year specifically trained veterinary student.

## Methods

### Study design

The present study includes a combination of prospective and retrospective investigations, where the prospectively collected auscultation findings from the veterinary cardiology specialist and the trained student were compared to auscultation findings recorded by first opinion veterinary practitioners. This study design is intentional in order to assess murmur documentation in first opinion veterinary practices.

### Animals

Between October 2015 and June 2016, 359 client-owned, apparently healthy puppies from 80 different litters were brought to the authors’ clinic by their breeders for voluntary screening for congenital portosystemic shunts using venous blood ammonia concentration measurement. Puppies were included in the present study if they were less than 90 days old when they were auscultated by a veterinary cardiology specialist (VSz) and when their health certificates (i.e. pet passports) were available. Exclusion criteria were puppies older than 90 days of age and the absence of written documentation in the puppy’s health certificate regarding the presence or absence of a cardiac murmur by a first opinion veterinary practitioner. In order to establish that the puppies were healthy, the veterinary cardiology specialist conducted a short owner interview and performed a focused clinical examination.

### Auscultation

All owners signed an informed consent. Each puppy was placed on a table in a quiet examination room and was identified by its microchip number. Auscultation was performed in a standing position on the left and right hemithorax. After auscultation on the left apical and basal cardiac regions, the right apical and basal regions were examined, and finally the left side was auscultated for a second time [2]. When a murmur was detected, its intensity (on a scale of 1–6, with 1 being the softest), the point of maximal intensity (left or right cardiac base or apex), the timing (systolic and/or diastolic, or continuous) and its additional character, such as musical or intermittently audible, were described [1, 2]. A murmur was classified as grade 1, if it was very soft, focal and was heard only after several seconds of careful auscultation; while a murmur was classified as grade 2 if it was soft, focal, but was heard immediately [1–4]. A murmur was defined as intermittent if a soft (1 or 2 out of 6) murmur was heard for the first time on the left hemithorax, but either it disappeared while the auscultation was still ongoing on the same anatomical location or it could not be identified when the left hemithorax was auscultated again [2–4]. Musical character of the murmur was defined as a soft, low- to medium-pitched vibrating sound [2, 5].

Puppies were auscultated by both the veterinary cardiology specialist and the student within a 1-h interval. Both investigators were blinded to each other’s findings and to the findings of the first opinion veterinary practitioner until completion of the auscultation of all the puppies on that day. Heart rates were not recorded. Both investigators used an acoustic stethoscope with a membrane diaphragm. The veterinary cardiology specialist’s stethoscope had a diaphragm diameter of 30 mm and the student’s diaphragm was 45 mm. The reasons for using different stethoscopes were personal preference, logistics and hygiene.

Prior to participation in the study, the student had received additional training in cardiac auscultation from the veterinary cardiology specialist. For this purpose she auscultated 62 puppies together with the veterinary cardiology specialist using her own acoustic stethoscope and an electronic stethoscope (with a recording possibility) on three separate days, approximately 1 h per day, in the first 3 weeks of October 2015. The veterinary cardiology specialist found a murmur in 8 of these puppies. The following breeds were represented in this subset of dogs: 18 Cairn terriers, 10 Irish wolfhounds, 8 Barbets, 7 Bernese mountain dogs, 7 Jack Russell terriers, 7 Yorkshire terriers and 5 Pugs. All but 3 of these puppies (all Yorkshire terriers) were included in the study population, as both the veterinary cardiology specialist and a first opinion veterinary practitioner auscultated them.

The pet passports were examined to assess if the first opinion practitioners had recorded abnormal auscultation findings. These data were collated after completing all the examinations for that day. Clinical records from the first opinion veterinary practitioners were not available, as none of the puppies had been referred.

After completion of the data analysis, the veterinary cardiology specialist phoned each veterinary practice that was involved in the health checks of at least 3 dogs in which a cardiac murmur was noted by the veterinary cardiology specialist but not by the first opinion veterinary practitioner. The telephone interviews were conducted in order to establish documentation routines during the physical examination. The first opinion veterinarians were asked whether or not they would routinely record the presence of soft murmurs on the pet passports even if the murmurs were suspected to be innocent. The telephone interviews with each veterinarian followed the same structure.

### Statistical methods

A commercially available statistical software package (SPSS 24.0, IBM Corp., Chicago, IL, USA) was used for data analysis. A P-value of less than 0.05 was considered to be significant. The age of the dogs is reported in days as median and range.

The degree of inter-observer agreements were evaluated by paired comparisons between the veterinary cardiology specialist, the first opinion veterinary practitioners and the veterinary student using Cohen’s kappa (ϰ). Agreement was regarded as: ‘poor’ ϰ ≤ 0.20, ‘fair’ 0.21 ≤ ϰ ≤ 0.40, ‘moderate’ 0.41 ≤ ϰ ≤ 0.60, ‘substantial’ 0.61 ≤ ϰ ≤ 0.80 or ‘good’ > 0.80.

## Results

From the 359 puppies, 28 were excluded from the study because 1 was too old (262 days), 1 had no age recorded, 7 had not been auscultated by a first opinion veterinary practitioner and 19 were not auscultated by the veterinary cardiology specialist. A total of 331 puppies from 71 litters, belonging to 10 different breeds, were enrolled. The following breeds were represented: 214 Cairn terriers (65%), 26 Dachshunds, 22 Bernese mountain dogs, 16 Jack Russell terriers, 11 Yorkshire terriers, 10 Irish wolfhounds, 10 Nova Scotia duck tolling retrievers, 9 Norfolk terriers, 8 Barbets and 5 Pugs. The Cairn terriers belonged to 51 litters and the other breeds belonged to 20 litters.

The median age of the 331 dogs at the time of auscultation at the authors’ clinic was 53 days (range 45–76 days). The median age of the same dogs when the auscultation was performed by a first opinion veterinary practitioner was 45 days (range 34–69 days), on average 9 days younger. First opinion veterinary practitioners from 43 different veterinary practices filled in the health certificates of the puppies. In most instances only the name of the veterinary practice, but not the veterinarian’s name, was identifiable in the passports. Because multiple veterinarians were employed at the majority of the practices, the number of veterinarians involved was higher than the number of practices. The exact number is unknown because the signatures were illegible. There was no further information available about the circumstances of the auscultation performed by the first opinion veterinary practitioners; only the presence or absence of a murmur was noted.

The veterinary cardiology specialist detected a murmur in 97 of the 331 puppies (29.3%). The median age of the puppies with a murmur was 53 days (range 45–76 days). Of the 214 Cairn terriers, 80 (37%) had a murmur, whereas only 17 of the 117 remaining auscultated dogs (15%) had a murmur. All murmurs were systolic and the murmur in 82 puppies (85%) had a musical character. The murmur intensity was grade 2 in 38 puppies and grade 1 in 59 puppies. Of the latter group, the murmur was intermittently audible in 14 puppies. The point of maximal intensity was at the region of the left cardiac base in 88 dogs (91%).

In the passports of the 331 puppies, only 2 dogs had a murmur noted. A murmur was documented by first opinion veterinary practitioners in only 1 of the 97 puppies (1%), in which the veterinary cardiology specialist heard a murmur (Table 1). In the other puppy whose passport recorded the presence of a murmur, the veterinary cardiology specialist did not hear a murmur (Table 1). When the results of the veterinary cardiology specialist and the first opinion veterinary practitioners were compared using Cohen’s kappa, a poor agreement (ϰ = 0.01) and a significant difference (P < 0.001) were found.

**Table 1:** Results of a veterinary cardiology specialist (cardiologist) and various first opinion veterinary practitioners (practitioners) on the presence or absence of a cardiac murmur in 331 clinically healthy puppies

	Practitioners		
	No murmur	Murmur	Total
Cardiologist			
No murmur	233	1	234
Murmur	96	1	97
Total	329	2	331

Of the 331 puppies, 252 were also auscultated by the trained student. In 66 of these puppies, a murmur was detected by her. When the results of the student and the veterinary cardiology specialist were compared, a fair agreement (ϰ = 0.40) with a significant difference (P = 0.024) was found (Table 2). Comparing the results of the student and first opinion veterinary practitioners, a poor agreement (ϰ = 0.03) with a significant difference (P < 0.001) was found (Table 3).

**Table 2:** Results of a veterinary cardiology specialist (cardiologist) and a trained final year veterinary student (student) on the presence or absence of a cardiac murmur in 252 clinically healthy puppies

	Student		
	No murmur	Murmur	Total
Cardiologist			
No murmur	145	23	168
Murmur	41	43	84
Total	186	66	252

**Table 3:** Results of various first opinion veterinary practitioners (practitioners) and a trained final year veterinary student (student) on the presence or absence of a cardiac murmur in 252 clinically healthy puppies

	Practitioners		
	No murmur	Murmur	Total
Student			
No murmur	186	0	186
Murmur	64	2	66
Total	250	2	252

A number of murmur characteristics were compared between the student and the veterinary cardiology specialist for the 252 dogs that were auscultated by both investigators. There was complete agreement about the timing of the murmurs between both the observers; all murmurs were recorded as systolic. The point of maximal intensity differed between the two observers in only 1 of the 43 dogs: the veterinary cardiology specialist noted that the point of maximal intensity was the right cardiac base and the student noted the left cardiac base. The findings of the two observers on the intensity of the murmurs showed much more variability, which is shown in Table 4. The most important differences on this aspect were that the student did not classify any murmur as being intermittent. The other striking difference was that in 30 puppies where the veterinary cardiology specialist detected a murmur, the student recorded no murmur. Finally, in 13 puppies where the veterinary cardiology specialist found a grade 2 murmur, the student recorded the murmur as grade 1.

**Table 4:** Differences in the results between the veterinary cardiology specialist (cardiologist) and the last year veterinary student on murmur intensities of the 84 puppies in which the veterinary cardiology specialist detected a murmur

	Student			
	No murmur	Intermittent murmur	1/6	2/6
Cardiologist				
Intermittent murmur	12	0	2	0
1/6	19	0	21	0
2/6	11	0	13	6

1/6 = murmur intensity of 1 out of 6

Ten first opinion veterinary practices were identified that performed the health checks of at least three dogs in which a cardiac murmur was noted by the veterinary cardiology specialist but not by the first opinion veterinary practitioner. Veterinarians from these ten first opinion veterinary practices performed the cardiac auscultation on 43 of the 96 puppies (45%) in which the cardiologist detected a murmur, but in their passports no murmur was noted. In the telephone interview, eight of the ten veterinarians said that they would always record the presence of a cardiac murmur in the puppies’ passports regardless of the murmur intensity. However, there were two veterinarians who admitted that they do not always make a note about the presence of a soft murmur in the passport if they thought that it was innocent.

## Discussion

The present study found a poor agreement between the documented auscultation findings of a veterinary cardiology specialist and first opinion veterinary practitioners regarding the presence of (soft) cardiac murmurs in 331 clinically healthy puppies. A cardiac murmur was documented by a first opinion veterinary practitioner in only 1 of the 97 puppies, in which the veterinary cardiology specialist detected a murmur. On the other hand, there was only 1 puppy where the first opinion veterinary practitioner noted a murmur, but the veterinary cardiology specialist did not hear a murmur. This latter puppy had either a non-pathological murmur that disappeared spontaneously between the two moments of auscultation or the stress level (and therefore the heart rate) on the two occasions was different, making the murmur inaudible on the second occasion.

There are a number of possible explanations for the discrepancy between the auscultation findings of the veterinary cardiology specialist and the first opinion veterinary practitioners, including: (1) first opinion veterinary practitioners did not recognise the soft murmurs, (2) the health certificates of the pet passports were filled in without the first opinion veterinary practitioners having performed an auscultation on the puppies, (3) the first opinion veterinary practitioners did perform the auscultation and did hear the murmurs, but they did not note them in the puppies’ passports, (4) the puppies were auscultated by the first opinion veterinary practitioners, but the murmurs were absent at that time, or (5) the veterinary cardiology specialist overanalysed the presence of murmurs.

A possible reason why first opinion veterinary practitioners less frequently noted a cardiac murmur in the present study could be that they have less time to devote to cardiac auscultation than a veterinary cardiology specialist, and a murmur with a maximal intensity of grade 1 may not be immediately noticed [1]. Time pressure can also mean that no efforts were made to look for a quiet room, if one was available. Pretest probability of finding a pathological murmur in this population is very low, because congenital cardiac anomalies have a low prevalence in the general canine population [7]. However, screening for congenital cardiac anomalies with a stethoscope is a quick, inexpensive and sensitive test, which would lead to documentation of the more prevalent non-pathological cardiac murmurs [2]. The prevalence of pathological murmurs caused by congenital cardiac anomalies in a general, non-referred canine population was reported to be 0.1% based on a population of 76,301 dogs [7], whereas the prevalence of non-pathological murmurs in puppies at the age of 7–8 weeks was reported to be 15–31% [2, 3]. The results of the telephone interview with the first opinion veterinary practitioners suggest that either the murmur was inaudible at the time of their auscultation, or they did not detect them. Though pet passports are official documents, they might not be the correct source of information, as the clinical records of the dogs might contain different information. Although differentiating non-pathological from pathological murmurs can be extremely challenging, if not impossible by auscultation, especially in cases of soft systolic murmurs, it is of major importance to perform the auscultation according to a systematic approach to detect local murmurs, as well as to document each cardiac murmur on the health certificate (i.e. pet passport) of the puppy at the first veterinary health check. In the country in which the study was performed, the pet passport is the only official document about the health status of the puppy that is available for the new owner when the breeder sells the puppy.

A minority of interviewed first opinion veterinary practitioners admitted that they would not always document the presence of a soft murmur in the health certificate of the puppy. There are two possible reasons for this behaviour: (a) assuming that the murmur is non-pathologic or (b) wanting to avoid conflict with the owner, who is typically the breeder at the time of the first health check.

The murmurs in the present study were presumed to be non-pathologic, however no additional tests, such as echocardiograms, were performed to establish the origin of the murmurs, as it was not an aim of the study. Based on human studies and our previous studies, all murmurs in the presented group were thought to be innocent [1–6]. This assumption is supported by the fact that the prevalence of soft cardiac murmurs in the population of the present study (29%) was comparable to the prevalence of innocent murmurs of our earlier studies (28% and 31%) performed on populations with very similar characteristics [2, 3]. Moreover, 20 Cairn terriers from the present cross-sectional study participated in a longitudinal study where they were followed up at approximately monthly intervals [4]. The murmurs in all 20 puppies disappeared spontaneously [4]. In our experience, non-pathological murmurs in puppies have auscultation characteristics similar to the most common type of non-pathological murmur in children, the “Still’s murmur” [2–6]. Non-pathological murmurs in clinically healthy puppies are always soft (1–2 out of 6) and systolic, and the majority have a low- to medium-pitched musical (vibrating) character [2–4]. According to fairly recently published guidelines on incidentally detected murmurs in dogs, non-pathological murmurs can be classified either as functional or innocent [1]. In both cases no structural heart disease is present. The difference between the two types of murmurs is that for the functional murmur a plausible physiologic explanation can be found (such as anaemia), whereas for innocent murmurs, no physiologic explanation can be identified [1]. The genesis of non-pathological murmurs in the dog is poorly documented. In children, non-pathological (accidental) murmurs are thought to arise from turbulent flow in the aorta [5, 6]. In puppies, physiological anaemia has been described as one of the contributors of the genesis of innocent murmurs, as lower blood viscosity can cause turbulent flow at lower velocity, presumably in the aorta [1, 2]. With this aetiology of physiological anaemia, the above described veterinary classification of functional versus innocent murmur is difficult to apply to non-pathological murmurs in healthy puppies.

The murmur intensity in the present population did not exceed 2 out of 6. Very soft and especially intermittently audible murmurs can be missed even by veterinary cardiology specialists who are less experienced in specifically searching for innocent murmurs in young puppies, as shown in our previous studies [2]. Failure to recognise innocent murmurs does not have negative consequences for the puppies [1, 2]. However, a potential danger of missing soft cardiac murmurs is that a louder, possibly pathological murmur could be classified as a soft, and probably non-pathological, murmur, and consequently no referral to a veterinary cardiology specialist would be recommended to the breeder or the new owner. To explore this hypothesis, more studies are needed. Missing a non-pathological murmur is not clinically important, however missing a pathological murmur could be detrimental for the individual puppy with emotional and financial sequelae for the breeder and the new owner, as well as legal consequences for the breeder and for the first opinion veterinary practitioner. In addition, using a standardised systematic auscultation protocol, i.e. listening in the left and right apical and basal cardiac areas, is essential to detect non-pathological as well as pathological murmurs. Despite applying a systematic auscultation protocol, the interpretation of auscultation findings can still remain subjective [8–10]. Whether or not the first opinion veterinary practitioners, whose results were assessed in the present study, used a standardized systematic auscultation protocol remains unknown.

The final year veterinary student, who was trained to recognise soft cardiac murmurs in puppies immediately before the start of the study (in a total of 3 h on 3 separate days with weekly intervals), had a better agreement with the veterinary cardiology specialist than first opinion veterinary practitioners in identifying soft murmurs. Various human and veterinary studies have investigated inter-observer variability and the effect of teaching on recognising cardiac murmurs [8–16]. The majority of these studies describe a high inter-observer variability [8–10] and conclude that training has a positive effect on detecting murmurs, especially those with a low intensity [11–13, 15–17]. One of these studies investigated the inter-observer variability of six veterinarians with different levels of experience in auscultation on recognising soft systolic murmurs in Cavalier King Charles spaniels caused by mitral valve insufficiency [8]. The degree of agreement was found to be poor [8]. Another study group compared the auscultation skills of ten internal medicine specialists, ten first opinion veterinary practitioners and ten veterinary students in describing murmur characteristics on recorded equine heart sounds [9]. High inter-observer variability was found, where only the specialists were able to provide correct descriptions [9]. In a different study, six veterinarians with different levels of experience in cardiac auscultation examined 27 Boxer dogs with and without a murmur [10]. Variable levels of agreement (Cohen’s kappa of 0.14–0.75) ranging from poor to substantial was found, with a positive correlation with the level of experience [10]. It has been shown in a human study that even a 1-h long online teaching session resulted in a significant improvement of auscultation skills [16]. Another human study emphasises the importance of repetition (i.e. practice), which is in line with our previous experience (i.e. learning curve) [2, 12]. Again another human study confirmed that training led to improved detection of soft, non-pathological murmurs in particular [15]. In children, the observers’ experience and training were shown as important factors that played a role in differentiating innocent from pathologic murmurs [5, 6]. The use of electronic stethoscopes has been shown to have a positive effect in teaching auscultation skills [17]. This tool was utilised in the training of the student of the present study too. However, the type of stethoscope does not seem to have a documented positive effect in identifying auscultation abnormalities [14].

### Limitations

An important weakness of the present study is that the auscultation findings of one veterinary cardiology specialist was used as gold standard. However, this study design is not unusual, as in a similar study with children the findings of a single paediatric cardiologist were used as the gold standard [5]. Our previous study on puppies with non-pathological murmurs showed that an analysable phonocardiogram could only be recorded in 71% of the population, therefore phonocardiography could not be used as a gold standard [3]. Including another veterinary cardiology specialist in the study would have resulted in more objective findings, as the two specialists could have acted as each other’s controls. Unfortunately, at the time of the study only one veterinary cardiology specialist was employed at the authors’ institution.

Auscultation by the veterinary cardiology specialist and the first opinion veterinary practitioners took place on different days and in different locations. The possible importance of these variables is that stress level of the dogs might have been different, which is thought to influence the development of soft cardiac murmurs because of changes in heart rates [1, 10, 18]. First opinion veterinary practitioners auscultated the puppies on average 9 days before the veterinary cardiology specialist and the trained student. Because lower haematocrit is known to contribute to the genesis of non-pathological cardiac murmurs in puppies, the prevalence of soft (innocent) cardiac murmurs should theoretically be at least as high or even higher at a younger age, since (physiological) anaemia has been shown to be less common with increasing age [1, 2, 19]. Therefore, the discrepancy between the findings of the veterinary cardiology specialist and the first opinion veterinary practitioners was less likely the result of a possibly different prevalence of cardiac murmurs at the two time points; however, this possibility cannot be ruled out. The different size of the puppies at the two time points might have had an effect on the different auscultation findings too. On the other hand, the authors hypothesise that the reason for higher prevalence of non-pathological murmurs in the Cairn terrier breed compared to other breeds is probably the small size of this breed since thicker thoracic walls of large-breed dogs may dampen the soft murmurs to an inaudible level [2]. The greater degree of agreement between the trained student and the veterinary cardiology specialist could potentially be due to the fact that they performed the auscultation in the same location and within a 1 h interval. Therefore, the conditions, which might influence the presence of non-pathological cardiac murmurs, were more similar, compared to the conditions of the first opinion veterinary practitioners. Despite the above mentioned shortcomings, the design of the present study was intentional in order to assess murmur documentation in first opinion veterinary practices in the field in a realistic manner. Likewise, an inter-observer variability study on schoolchildren in identifying innocent cardiac murmurs by a single paediatric cardiologist and several school medical officers had a comparable study design as that of the present study [5]. That study had a very similar time interval between the two examinations (namely 2 weeks), and also showed a high inter-observer variability [5].

The discrepancy between the auscultation findings of the veterinary cardiology specialist and the student, as well as that between the veterinary cardiology specialist and the first opinion veterinary practitioners, could also be the result of the different type of stethoscopes with different sizes of membrane used. However, in humans the type of equipment and the diaphragm size has not been shown to influence the precision of auscultation [13, 20].

Another limitation of the present study is that the puppies’ clinical records were not available for review. It is possible that the presence of a murmur was noted in the clinical record of the first opinion veterinary practice, but not in the health certificates (i.e. pet passports) of the puppies.

Finally, awareness bias could have played a role in the different findings between the first opinion veterinary practitioners and the veterinary cardiology specialist. The veterinary cardiology specialist and the student were both aware of their participation in a study concerning cardiac murmurs, however the first opinion practitioners were not. If someone is actively searching for a murmur they are more likely to find one. On the other hand, in an ideal situation, each examination in the field should be performed as if the investigator was participating in a study.

## Conclusions

The present study shows that soft cardiac murmurs are rarely documented during the first veterinary health check in clinically healthy puppies by first opinion veterinary practitioners. The most likely explanation for this finding is that the soft murmurs were not recognised. Though soft cardiac murmurs may not be clinically relevant, finding them is evidence of a carefully performed auscultation. Missing an innocent murmur is not of great clinical importance, but missing a pathological murmur could prove detrimental for the individual puppy. Finally, we conclude that auscultation skills can be learnt and improved by education and by adopting a systematic and thorough approach.

## Data Availability

The datasets used and/or analysed during the current study are available from the corresponding author on reasonable request.
